# Clinical Evaluation for Space Maintainer after Unilateral Loss of Primary First Molar in the Early Mixed Dentition Stage

**DOI:** 10.1155/2021/3967164

**Published:** 2021-12-27

**Authors:** Shahram Mosharrafian, Ali Baghalian, Mohammad Hassan Hamrah, Mojgan Kargar

**Affiliations:** ^1^Department of Pediatric Dentistry, School of Dentistry, Tehran University of Medical Sciences, Tehran, Iran; ^2^Department of Pediatric Dentistry, School of Dentistry, Shahid Sadoughi University of Medical Sciences, Yazd, Iran

## Abstract

*Background and Objectives*: Controversy exists regarding the need for a space maintainer after early unilateral loss of a primary first molar. This study aimed to assess the need for a space maintainer after unilateral loss of a primary first molar in the early mixed dentition period. *Materials and Methods*. In this cross-sectional study, fifty children between 6 and 8 years who had lost a primary first molar unilaterally later than 6 months ago were randomly selected. Midline deviation, molar and canine relationships at both sides, facial growth pattern, and the amount of space loss were all assessed. Data were analyzed using SPSS version 25 via the one-sample *t*-test, paired *t*-test, and linear regression (alpha = 0.05). *Results*. The mean amount of space loss was 1.36 ± 0.78 mm (1.32 mm in the maxilla and 1.40 mm in the mandible). Time since tooth extraction and facial pattern had significant correlations with space loss (*P* < 0.05). *Conclusion*. In this particular age group, it is imperative to precisely assess the related factors such as the facial pattern and time since tooth extraction to decide about the placement of a space maintainer for a prematurely lost primary first molar.

## 1. Introduction

Exfoliation of primary teeth and eruption of permanent teeth are normal physiological processes that often occur simultaneously at a specific age. However, premature loss of primary teeth may occur in some cases for several reasons [1]. In many cases, premature loss of primary teeth may impair the integrity and uniformity of dental arch and adversely affect the future leveling and alignment of permanent teeth. Early tooth loss can result in overeruption of the opposing teeth, mesial drifting of the teeth located distal to the lost tooth, and distal drifting of the teeth located mesial to the lost tooth [2]. Asymmetry of the dental arch may also occur [3]. Mesial drifting of the teeth located distal to the lost tooth is greater following the loss of a primary second molar than a primary first molar. Distal drifting of the teeth located mesial to the lost tooth is more common following the loss of a primary first molar compared with a primary second molar [4].

In cases in whom early extraction/loss of primary teeth due to extensive caries or other reasons is inevitable, placement of a space maintainer is the safest option for space maintenance in the dental arch [1].

Although not much controversy exists regarding the need for placement of a space maintainer after primary second molar loss, the need for space maintenance and clinical management of space after early loss of primary first molar is a highly debated topic [5]. In general, there is a consensus that early loss of the primary mandibular first molar mainly results in distal drifting of the primary mandibular canine, while in the maxilla, mesial drifting of the primary second molar into the extraction space is more common. The majority of studies on this topic have reported space loss almost always after early loss of primary first molars; however, the amount of space loss and the need for its clinical management are still matters of controversy [6–13].

Early loss of primary first molars requires placement of a space maintainer only in some certain circumstances. In the process of active eruption of permanent first molars between 5 to 7 years of age, mesial forces may result in loss of the primary first molar space. Moreover, space loss may occur when permanent first molars have an end-to-end relationship before the early or late mesial shift occurs [14].

The role of facial morphological differences of patients (vertical or horizontal growth pattern) in space loss is often overlooked. Alexander et al. [14] demonstrated greater space loss in children with hyperdivergent facial pattern (high facial height) compared with hypodivergent patients (low facial height) [14].

According to the literature, space loss in the mandible is greater than that in the maxilla. Also, space loss as the result of primary second molar extraction is greater than that following primary first molar loss. Furthermore, space loss is greater when the teeth are lost at a younger age and also in patients with dental crowding compared with those with dental spacing [5]. Ronnerman and Thilander believed that early exfoliation of primary first molars has a small role in dental crowding [5].

It has been discussed that large dental arches with normal or excessive space are not affected by early loss of primary teeth. On the other hand, the extraction space is probably closed and leads to crowding of permanent dentition in small primary dental arches with space shortage [7].

Since comprehensive assessment of all possible factors with probable effects on the amount of space loss following early loss of primary first molars in the early mixed dentition period has not been performed, this study aimed to comprehensively assess all possible factors in this regard and evaluate the need for a space maintainer after unilateral loss of a primary first molar in the early mixed dentition period to prevent unnecessary placement of space maintainers.

## 2. Materials and Methods

This cross-sectional study evaluated 50 children in the early mixed dentition period that had lost a primary first molar unilaterally later than (before) 6 months ago (25 in the maxilla and 25 in the mandible). The inclusion criteria for the children were as follows:

- Not using any appliance or space maintainer since the loss of their primary first molar
- Age group of patients between 6 years and 8 years
- All teeth, except for the unilaterally lost primary first molar, had to be present in the dental arch, and no other missing teeth resulting from extraction or trauma
- Absence of interproximal caries or their optimal-quality restoration
- Absence of jaw asymmetry or functional mandibular shift
- Parents consented to the participation of their children in the study

The following parameters were recorded in clinical examination of patients:

- Age of the child
- Time of primary tooth loss by asking the parents
- Type of molar and canine relationships in the right and left sides
- Facial pattern
- Degree of midline deviation in the affected jaw relative to the facial midline

**Figure 1:**
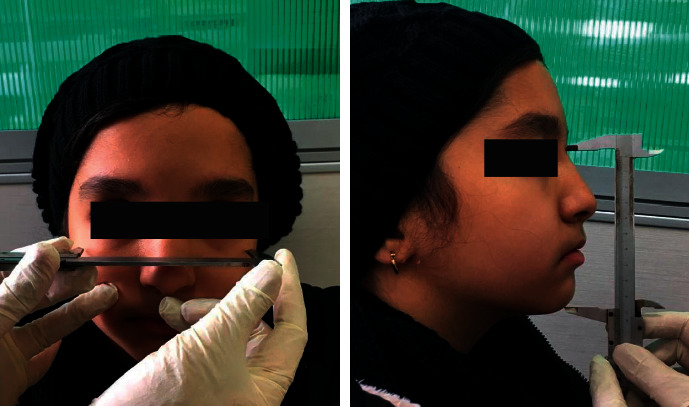
Measuring the facial width and height using a caliper.

The Hall and Farkas method was adopted to determine the facial growth pattern of the patients [15, 16]. For this purpose, the bizygomatic width to the maximum facial height ratio was calculated, and the facial growth pattern was determined as such. If the obtained value was ≤0.75, the facial pattern was classified as leptoprosopic; if the value was 0.76–0.79, the facial pattern was classified as mesoprosopic, and if it was ≥0.8, the facial pattern was classified as euryprosopic (Figure 1).

To assess the dental midline deviation relative to the facial midline, the nasion and pogonion points were connected by a dental floss, and this line served as the facial midline. The midline between the two central incisors of the respective jaw was compared with the facial midline, and in case of deviation, its amount was recorded.

**Figure 2:**
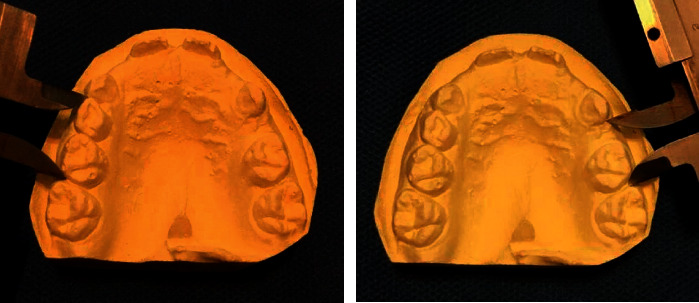
Measuring the distance between the distal surface of the primary canine and mesial surface of the permanent first molar on dental casts by using a caliper.

An alginate impression was made from the involved jaw, and a cast was poured. The distance between the midpoint of the mesial surface of the permanent first molar and midpoint of the distal surface of the primary canine in both right and left quadrants was measured by a caliper. The difference in values between the two quadrants indicated the amount of space loss (Figure 2).

The degree of dental crowding was quantified on the study cast by using the Little irregularity index.

The collected data were statistically analyzed using SPSS version 25. The mean, standard deviation, frequency, and percentage of the values were calculated and reported. Data were analyzed using one-sample *t*-test (to confirm space loss). The paired *t*-test was applied to compare the distance between the canine and permanent first molar in the extraction and control quadrants. The linear regression test was applied to analyze the effect of different factors on space loss. The level of significance was set at 0.05.

A sample size of 50 was determined at an alpha of 0.05 and a power of 0.95 using software PASS11.

## 3. Results

Fifty children between 6 and 8 years were evaluated in this study, of which 25 had lost a maxillary primary first molar unilaterally and 25 had lost a mandibular primary first molar unilaterally.

Table 1 presents the molar and canine relationship in the right and left sides. The lost primary first molar was in the right side in 30 (60%) and in the left side in the remaining patients. Of all, 31 (62%) did not have midline deviation and 20 (40%) had dental crowding.

Table 2 presents the age, facial skeletal pattern, midline deviation, and space loss. As shown, the mean age of children was 7.30 ± 0.67 years, and the mean percentage of facial width to facial height ratio was 0.81% ± 0.05%. The time since the extraction was 13.54 ± 6.28 months. The mean amount of space loss was 1.36 ± 0.78 mm (1.32 mm in the maxilla and 1.40 mm in the mandible). Assessment of different facial patterns revealed that the euryprosopic pattern had the highest frequency (60%).

Leptoprosopic facial growth pattern (mean value of 2.46 mm): the mean space loss was smaller in the mesoprosopic (mean value of 1.84 mm) and euryprosopic group (mean value of 0.94 mm) Table 3.

Table 4 compares the distance between the central incisor and primary canine in the right and left quadrants of the maxilla and mandible. As shown, this distance in the extraction quadrant was significantly greater than that in the control quadrant in the mandible (*P*=0.004). However, this difference was not significant between the two quadrants in the maxilla (*P*=0.649).

Table 5 shows the molar relationship in the right and left sides. As indicated, the class II molar relationship was the most stable and remained unchanged in 100% of the cases, while the class I molar relationship was the least stable and remained unchanged in 68% of the cases.

Table 6 shows the canine relationship in the extraction side and control side. As indicated, the class II canine relationship was the most stable and remained unchanged in 100% of the cases, while the class I canine relationship was the least stable and remained unchanged in 66% of the cases (was the same in both quadrants).

Multivariate analysis by the backward linear regression model was then performed. In this model, space loss was the dependent variable, and the involved jaw, age, dental crowding, molar, and canine relationship in the control quadrant, facial pattern, and time since extraction were the independent variables. The results showed that time since extraction and facial pattern had significant correlations with space loss. In other words, the longer the time since extraction, the greater the space loss (*P*=0.032), and the greater the facial width to facial height ratio, the smaller the space loss would be (*P* < 0.001). Other variables had no significant correlation with space loss (*P* < 0.05) (Table 7).

## 4. Discussion

Although not much controversy exists regarding the need for placement of a space maintainer after primary second molar loss, the need for space maintenance and clinical management of space after early loss of a primary first molar is a highly debated topic. Thus, this study was carried out to address this topic and evaluate the role of different factors in this respect. The effects of age of the child, time since extraction, involved jaw, molar and canine relationship, facial pattern, and anterior crowding on space loss were evaluated. The mean amount of space loss was 1.36 mm in this study; this value was greater in the mandible (1.40) than in the maxilla (1.32 mm) but not significant. Lin et al. [17] demonstrated 1.26 mm space loss at 6 months following unilateral loss of a maxillary primary first molar in 4-to-7-year-old children. The age range of children and the amount of space loss in the present study were close to the values reported by Lin et al. [17]. Of the tested variables, multivariate analysis by linear regression showed significant correlations of time since extraction and facial pattern of patients with space loss. In the present study, the longer the time since tooth extraction, the greater was the space loss (*P*=0.032) such that in patients that 24 months had passed since their tooth extraction, the space loss was averagely 1.79 mm. Evidence shows that maximum space loss occurs in the first 6 months to 1 year after tooth extraction [2, 7, 18]. The current results also showed maximum space loss in the first 6 to 9 months after tooth extraction (averagely 1.22 mm). Moreover, the present results indicated that as the time since extraction increased, the space loss significantly increased as well. This finding may be due to the specific age group of children in the present study since space loss is also affected by the eruption of permanent first molars. Also, in this stage, some other teeth are erupting in addition to permanent first molars, and probably occupy the available space.

The present results also indicated maximum space loss in patients with leptoprosopic facial growth pattern (mean value of 2.46 mm). The mean space loss was smaller in the mesoprosopic (mean value of 1.84 mm) and euryprosopic group (mean value of 0.94 mm). The linear regression analysis showed that the facial pattern significantly affected the amount of space loss such that by an increase in facial width to height ratio, the space loss significantly decreased. Alexander et al. [14] demonstrated greater space loss in children with hyperdivergent facial pattern (high facial height) compared with hypodivergent patients (low facial height), which was in agreement with our results. Multivariate analysis in the present study showed that other variables such as age, crowding, midline deviation, jaw, and molar or canine relationship had no significant effect on space loss. The degree of crowding according to Little's irregularity index had no correlation with space loss either.

Kumari and Kumari [7] evaluated 40 children between 6 and 9 years and reported that large dental arch with normal or excessive space was not influenced by early loss of primary teeth. On the other hand, space shortage and small primary dental arch would probably lead to closure of the extraction space and crowding of permanent dentition [7]. The present results were different from those of Kumari and Kumari [7]; thus, further investigations are required on the effect of crowding, especially in severe cases on space loss, and there is a need for a space maintainer in such cases.

In the present study, the amount of space loss was not significantly different between the maxilla and mandible (although it was slightly greater in the mandible). Nonetheless, several studies demonstrated that space loss in the mandible was more common than in the maxilla [5–8]. McDonald discussed that loss of upper and lower primary first molars results in almost equal space loss; the amount of space loss is usually influenced by the time since the extraction of the primary first molar [2]. The same results were obtained in the present study.

Comparison of the distance between the distal surface of the central incisor and the mesial surface of the canine tooth at both sides in the maxilla and mandible showed that this distance in the extraction quadrant was significantly greater than that in the control quadrant in the mandible, which indicates distal drifting of the primary canine in case of early loss of the mandibular primary first molar. However, the difference in this distance was not significant between the two quadrants of the maxilla. Lin and Chang & Lin et al. [8, 17] in their studies demonstrated the distal movement of the canine tooth following early extraction of the primary first molar both in the maxilla and mandible. Cuoghi et al. [13] indicated distal drifting of the canine tooth and space shortage following unilateral loss of the mandibular primary first molar, which was in agreement with the present results.

With regard to the molar relationship, the maximum and minimum space loss were recorded in cases with distal step and mesial step, respectively; however, the difference in this respect was not significant. Alexander et al. [14] showed minimum space loss in the hypodivergent and class I molar relationship in both the maxilla and mandible and the hypodivergent facial pattern and end-to-end molar relationship in the maxilla. In the present study, no significant difference was noted in space loss between different classes of molar relationship, which can be due to unequal frequency distribution of different classes of occlusion in our study population. Thus, further studies with larger sample size are required to assess the effect of the molar relationship on space loss.

Assessment of the role of factors such as crowding, molar and canine relationship, type of dental arch, age of the patient, and time since extraction in space loss was a strength of this study.

Cross-sectional design and inadequate sample size for some variables such as crowding and molar and canine relationship were among the limitations of this study. Future studies with larger sample size and longitudinal design are recommended to further assess the effect of different variables on space loss.

## 5. Conclusions

The space loss was averagely low in this study (1.36 mm). According to the results, in this particular age group, assessment of factors such as facial pattern and time since extraction is required after early loss of a primary first molar to decide about the need for placement of a space maintainer, and the results of this study indicated maximum space loss in patients with leptoprosopic facial growth pattern (mean value of 2.46 mm).

## Figures and Tables

**Table 1:** Extraction and nonextraction side with occlusion type.

	Occlusion type	Extraction side (*N*)	Extraction side (percentage) (%)	Control side (number)	Control side (percentage) (%)
Molar relationship	Class I	12	24	16	32
	Class II	13	26	10	20
	Class III	1	2	0	0
	Mesial step	13	26	13	26
	Distal step	8	16	7	14
	Flush terminal plane	3	6	4	8

Canine relationship	Class I	30	60	36	72
	Class II	13	26	8	16
	Class III	7	14	6	12

**Table 2:** Age, facial skeletal pattern, and space loss.

Variable	Minimum	Maximum	Mean	Std. deviation
Age (years)	6	8	7.30	0.678
Time since extraction (months)	6	24	13.54	6.28
Facial width/height ratio (mm)	0.74	0.96	0.81	0.05
Crowding (mm)	0	4.80	0.99	1.44
Midline deviation (mm)	0	2	0.51	0.71
Space loss (mm)	0.10	3.30	1.36	0.78

**Table 3:** Relationship between facial form and space loss.

Facial form	*N*	Percentage (%)	Means of space loss (mm)
Leptoprosopic	5	10	2.46
Mesoprosopic	15	30	1.84
Euryprosopic	30	60	0.94

**Table 4:** Distribution between the extraction and nonextraction side.

Distance		Mean	Std. deviation
Maxilla	Control side	5.5240	1.06312
	Extraction side	5.5840	0.87306

Mandible	Control side	4.7800	0.88882
	Extraction side	5.2330	0.58218

**Table 5:** Comparison of the molar relationship between the extraction and control sides.

Molar relationship in the control side		Molar relationship in the extraction side					
		Class I	Class II	Class III	Mesial step	Distal step	FTP
Class I	Percentage	68.8%	18.8%	6.3%	6.3%	0%	0%
	Number	11	3	1	1	0	0

Class II	Percentage	0%	100%	0%	0%	0%	0%
	Number	0	10	0	0	0	0

Mesial step	Percentage	7.7%	0%	0%	84.6%	7.7%	0%
	Number	1	0	0	11	1	0

Distal step	Percentage	0%	0%	0%	14.3%	85.7%	0%
	Number	0	0	0	1	6	0

Flush terminal plane	Percentage	0%	0%	0%	0%	25%	75%
	Number	0	0	0	0	1	3

**Table 6:** Comparison of the canine relationship between the extraction and control sides.

Canine relationship in the control side		Canine relationship in the extraction side		
		Class I	Class II	Class III
Class I	Percentage	83.3%	8.3%	8.3%
	Number	30	3	3

Class II	Percentage	0%	100%	0%
	Number	0	8	0

Class III	Percentage	0%	33.3%	66.6%
	Number	0	2	4

**Table 7:** The linear regression analysis for variables.

Model		Unstandardized coefficients		Standardized coefficients	*t*	Sig.
		*B*	Std. error	Beta		
1	(Constant)	10.976	1.102		9.964	0.000
	Teeth extraction duration	0.023	0.011	0.188	2.206	0.032
	Facial pattern	−12.121	1.321	−0.781	−9.172	0.000

## Data Availability

Data will be made available on request (contact information: kargar.mojgan@yahoo.com).
